# Developing and defining core climate and environmental epidemiology competencies for field epidemiology training programs in the Eastern Mediterranean Region: a Delphi study

**DOI:** 10.3389/fpubh.2026.1902013

**Published:** 2026-08-06

**Authors:** Yousef Khader, Muhammad Sartaj, Mohannad Al Nsour, Ruba Kamal Alsouri, Mirza Zeeshan Iqbal Baig, Ariana Zeka, Muhammad Asif Khan, Rebecca Mary Close

**Affiliations:** 1 Department of Public Health, Faculty of Medicine, Jordan University of Science and Technology, Irbid, Jordan; 2 UK Health Security Agency, London, United Kingdom; 3 Eastern Mediterranean Public Health Network (EMPHNET), Amman, Jordan; 4 Environmental Epidemiology, Radiation, Chemicals, Climate and Environmental Hazards Directorate, UK Health Security Agency, Chilton, United Kingdom; 5 Institute of Global Health, University College London, London, United Kingdom

**Keywords:** climate change, competency framework, Delphi method, Eastern Mediterranean Region, environmental epidemiology, field epidemiology training program, One Health, public health workforce development

## Abstract

**Background:**

Climate change, environmental degradation, and chemical hazards increasingly shape population health risks, particularly in the Eastern Mediterranean Region (EMR), where water scarcity, rapid urbanization, conflict, and fragile health systems amplify vulnerability. Despite these growing threats, Field Epidemiology Training Programs (FETPs) lack systematically defined competencies in climate change and environmental epidemiology. This study aimed to identify, prioritize, and validate a core set of practice-oriented competencies for integrating climate change and environmental epidemiology into FETPs, with particular relevance to the EMR.

**Methods:**

We conducted a three-round modified Delphi study. Round 1 used a qualitative online survey to elicit expert input on essential competencies, priority areas, and implementation considerations. In Round 2, experts rated synthesized competencies using a 5-point Likert scale to assess importance and curricular inclusion. Round 3 consisted of a multidisciplinary expert workshop to validate and refine the competencies, clarify their applicability across FETP levels (Basic, Intermediate, Advanced), and translate them into applied learning modalities, including teaching case studies and structured fieldwork activities.

**Results:**

Consensus was achieved on a comprehensive set of competencies spanning foundational knowledge; environmental exposures and risk assessment; epidemiological and analytical skills; climate-informed environmental public health surveillance; environmental incident and event response; vulnerability, adaptation, and preparedness; policy and governance; risk communication and community engagement; and One Health and multisectoral collaboration. The highest-rated competencies extended core field epidemiology functions—particularly surveillance, outbreak and environmental event investigation, and data analysis—into climate and environmental contexts. Workshop validation confirmed the relevance, clarity, and feasibility of all competency domains and emphasized tiered implementation and integration within existing FETP curricula.

**Conclusion:**

This study presents a consensus-based, practice-oriented competency framework for integrating climate change and environmental epidemiology into FETPs. By combining Delphi consensus with expert validation and operationalization, the framework offers a feasible and scalable approach to strengthening climate-resilient field epidemiology capacity, particularly in vulnerable regions such as the EMR.

## Introduction

The Eastern Mediterranean Region (EMR) bears a disproportionately high burden of environmental, chemical, and climate-related health risks, driven by rapid urbanization, water scarcity, fragile health systems, conflict, displacement, and limited environmental governance. Environmental risk factors account for a substantial proportion of preventable morbidity and mortality globally and in the EMR, with air pollution, unsafe water and sanitation, and chemical exposures among the leading contributors to disease burden (1). The EMR experiences particularly high exposure to ambient and household air pollution, with several countries consistently ranking among those with the highest particulate matter (PM₂.₅) concentrations worldwide (2). Environmental epidemiology, the branch of epidemiology concerned with studying the distribution and determinants of health outcomes in relation to physical, chemical, biological, and social exposures in the environment, provides the methodological foundation for identifying, quantifying, and responding to these exposure–outcome relationships.

Chemical hazards represent a critical and often under-recognized public health threat in the region. Countries in the EMR face recurrent risks related to industrial pollution, pesticide exposure, hazardous waste mismanagement, fuel combustion, and accidental or intentional chemical incidents. Empirical studies document elevated occupational and community exposure to pesticides, heavy metals, and industrial chemicals in multiple EMR settings, often in the absence of adequate regulatory oversight or surveillance systems (3, 4). In several EMR settings, protracted armed conflict has compounded these risks. Beyond the direct loss of tens of thousands of lives, war has caused widespread destruction of environmental, water, and sanitation infrastructure. In addition, the intentional use of toxic and explosive warfare has generated long-term environmental contamination and population exposures whose health consequences remain poorly characterized. The limited evidence base on these conflict-related environmental hazards underscores the imperative of building a workforce equipped to recognize, monitor, and investigate them. WHO assessments further highlight gaps in chemical event preparedness, poison control infrastructure, and routine environmental exposure surveillance across many countries in the region, limiting early detection and response to chemical hazards (5). Strengthening routine environmental exposure and health surveillance is therefore essential to monitor both the acute and long-term health impacts of these exposures and to support early detection and response, consistent with core capacities under the International Health Regulations (IHR).

Climate change is intensifying these existing environmental and chemical risks. The EMR is warming faster than the global average and is experiencing increasing frequency and intensity of heatwaves, prolonged droughts, water scarcity, and extreme weather events (6). Beyond intensifying hazards directly, climate change can act as a risk multiplier by mobilizing and redistributing environmental contaminants across land, water, and air through flooding and surface run-off, drought-driven dust, and the release of previously contained chemical stores, thereby altering population exposure patterns, particularly where inadequate infrastructure amplifies these environmental and climatic stressors. These climate hazards exacerbate risks of heat-related illness, dehydration, food insecurity, malnutrition, displacement, and conflict. They also alter the transmission dynamics of climate-sensitive diseases such as dengue, leishmaniasis, cholera, and other water-borne and vector-borne diseases (7).

The convergence of climate hazards, environmental degradation, and chemical risks creates complex exposure profiles that challenge traditional public health surveillance and response models. Increasingly, disease outbreaks and health emergencies are shaped by interacting environmental, climatic, and social determinants rather than single etiologic agents. This complexity underscores the need for integrated competencies in climate and environmental epidemiology, including exposure assessment, climate-informed surveillance, early warning systems, outbreak investigation incorporating environmental drivers, and effective multisectoral risk communication (8, 9).

Field Epidemiology Training Programs (FETPs) represent a cornerstone of global applied epidemiology capacity building through competency-based training, mentored fieldwork, and service-oriented learning. With more than 80 programs worldwide, FETPs play a central role in strengthening national surveillance, outbreak investigation, and emergency response capacities (10). However, despite growing recognition of climate change as a major public health threat, competencies related to climate change and environmental epidemiology are not yet systematically defined or institutionalized across FETP curricula. Existing integration varies widely by country and region and is often driven by *ad hoc* initiatives rather than standardized, competency-based frameworks (11).

In regions such as the EMR, where climate change, environmental degradation, chemical hazards, conflict, and displacement intersect, this gap has critical implications for preparedness and response. Strengthening climate change and environmental epidemiology competencies within FETPs is essential to equip frontline epidemiologists with the skills needed to anticipate, detect, and respond to increasingly complex and interconnected health threats. To address this gap, we conducted a three-round Delphi study to identify, refine, and achieve expert consensus on core competencies in climate change and environmental epidemiology for integration into FETPs. By drawing on multidisciplinary expertise and focusing on applied public health practice, this study aimed to provide a robust, consensus-based foundation to guide curriculum development and institutionalization of climate and environmental epidemiology within FETPs, with particular relevance for the EMR. Specifically, this study was guided by three research questions: (1) What core competencies in climate change and environmental epidemiology should be integrated into FETPs? (2) How should these competencies be prioritized and distributed across the Basic, Intermediate, and Advanced FETP levels? (3) How can these competencies be translated into applied, practice-oriented learning activities suitable for integration within existing FETP curricula, particularly in the EMR?

## Methods

### Study design

We conducted a three-round Delphi study to identify, refine, and achieve expert consensus on core competencies in climate change and environmental epidemiology for integration into FETPs. The Delphi technique was selected because it is well-suited for consensus building in emerging and interdisciplinary fields where empirical evidence is limited and expert judgment is required. Key Delphi principles were applied, including anonymized responses, iterative feedback, and structured refinement of items across rounds.

### Expert panel selection

Experts were purposively selected to ensure multidisciplinary expertise and geographic representation, with an emphasis on applied public health practice and workforce development. Eligibility criteria included: (1) professional experience in epidemiology, environmental health, climate change, toxicology, One Health, or public health policy; and/or (2) direct involvement in Field Epidemiology Training Programs (FETPs) as directors, resident advisors, coordinators, faculty members, or mentors; and/or (3) experience working within ministries of health, national public health institutes, academic institutions, or international and regional public health organizations.

Delphi Round 1 included 29 experts with diverse professional backgrounds. The majority of participants reported more than ten years of professional experience. Experts were invited via email, and participation was voluntary. Panel members were identified from the FETP and EMPHNET regional networks and these initial experts were invited to nominate additional qualified colleagues. Of the 29 experts who completed Round 1, 30 participated in Round 2 (one additional nominated expert joined at Round 2), and 21 took part in the Round 3 validation workshop; the majority of Round 3 participants had contributed to at least one earlier round. Round 3 participation was limited to 21 participants because travel restrictions and logistical constraints affected workshop attendance.

### Data collection

Data were collected using online survey questionnaires administered in three sequential Delphi rounds. Survey links were distributed electronically via email, allowing participants to complete the questionnaires remotely at a time of their convenience. Online data collection enabled participation from experts across multiple countries and institutions and facilitated standardized administration of the survey instruments.

Responses were captured directly through the online survey platform and exported for analysis. No personally identifiable information was included in the analysis datasets.

### Delphi round 1: exploration and item generation

Delphi Round 1 employed an open-ended qualitative questionnaire designed to elicit expert perspectives on competencies required for climate change and environmental epidemiology training within FETPs. The Round 1 open-ended questions were developed by the study team based on a review of existing FETP core competencies and published climate- and environmental-health competency frameworks, and were pilot-reviewed internally for clarity before distribution. Participants were asked to identify:

- Core competencies in climate change and environmental epidemiology
- Priority competencies for FETP trainees
- Existing FETP competencies that could be adapted or expanded
- Relevant technical topics and field-based learning activities
- Barriers and enablers to institutionalizing climate and environmental epidemiology training
- Key stakeholders for curriculum development and implementation

Qualitative responses were analyzed using thematic content analysis. Two reviewers independently reviewed and coded responses and grouped them into competency domains and thematic categories. Discrepancies in coding were resolved through discussion and consensus. The resulting synthesized competency list informed the development of the Delphi Round 2 rating instrument. To illustrate the synthesis process, multiple free-text statements from Round 1—for example, “residents should be able to detect heat-related illness,” “integrate temperature and air-quality data into surveillance,” and “monitor vector-borne disease trends linked to rainfall”—were grouped under a single consolidated Round 2 item, “Monitoring and responding to climate-sensitive conditions (vector-borne, water-borne, heat-related).” Similar grouping of conceptually related statements produced each of the competency items, subsequently rated in Round 2.

### Delphi round 2: rating and consensus

Delphi Round 2 employed a structured online rating questionnaire derived directly from the synthesis of Round 1 findings. Participants were asked to assess each competency according to importance, rated on a 5-point Likert scale (1 = lowest importance; 5 = highest importance), and curricular inclusion, indicating whether the competency should be included in FETP curricula. Competencies were organized into thematic domains, including: foundational knowledge; environmental health exposures and risk assessment; epidemiological and analytical skills; environmental public health surveillance; environmental incident and event response; vulnerability and adaptation; policy and governance; risk communication; and One Health and intersectoral collaboration.

### Consensus criteria

Consensus was assessed descriptively based on mean Likert scale scores, response distributions, and patterns of agreement on curricular inclusion and appropriate FETP training levels. Competencies with consistently high mean ratings (mean ≥4.0) and broad participation across respondents were considered to demonstrate strong consensus for inclusion. These consensus criteria were specified *a priori*, before Round 2 data collection and analysis. In addition to mean ratings, we examined the proportion of respondents assigning a rating of ≥4 and the interquartile range (IQR) for each item as supplementary indicators of agreement and dispersion. Rather than applying strict exclusion thresholds, competencies were comparatively ranked to inform prioritization and level-specific placement within FETP curricula, reflecting the exploratory and consensus-oriented nature of the study. Strict exclusion thresholds were intentionally avoided to preserve emerging competencies that may be critical for future climate-related public health practice but are not yet uniformly institutionalized. Because no formal exclusion threshold was applied, no competency was removed solely on the basis of its Round 2 rating. The resulting list is intentionally comprehensive; programs are therefore encouraged to interpret it as a prioritized menu rather than a mandatory checklist. The relative ranking by mean rating, proportion of ratings ≥4, and IQR, together with the tiered (Basic/Intermediate/Advanced) placement described below, can be used to sequence adoption—beginning with the highest-consensus competencies that extend core FETP functions—according to each program’s context, capacity, and priorities.

### Delphi round 3

The workshop convened 21 multidisciplinary participants, including senior public health professionals, academics, and policy experts with expertise in public health, environmental health, and climate change. Participants were drawn from universities, ministries of health, FETPs, international public health networks, and national public health agencies. This diverse composition ensured a balance of academic, policy, and field-based perspectives, enabling discussions grounded in both applied public health practice and regional health priorities. The workshop participants partially overlapped with the Delphi panel: the majority had contributed to Round 1 and/or Round 2, ensuring continuity, while additional stakeholders—including representatives from ministries of health and environment, academic institutions, and regional public health organizations who had not taken part in the earlier rounds—were invited to broaden the range of implementation and policy perspectives during validation. The workshop employed facilitated plenary discussions, small-group thematic work, and structured consensus exercises. Participants reviewed the prioritized competency domains and statements from Round 2 and discussed their clarity, relevance, feasibility, and applicability across different FETP levels (Basic, Intermediate, Advanced). These tiers correspond to the established three-tier structure of the FETP network endorsed by WHO and the US CDC: the Basic (Frontline) tier targets surveillance officers at the local/district level and emphasizes timely detection, reporting, and initial response; the Intermediate tier serves mid-level epidemiologists at the subnational level and adds analytic investigation and program evaluation skills; and the Advanced tier prepares epidemiologists for national-level leadership, complex analysis, and applied research. Participants were also asked to propose illustrative teaching case studies and fieldwork activities that could be used to build the identified competencies in applied training settings. Consensus during Round 3 was assessed qualitatively through group agreement and synthesis across facilitated sessions rather than quantitative scoring. This approach was selected to support validation and implementation planning, consistent with the workshop’s focus on translating competencies into practice-oriented training approaches.

### Ethical considerations

The study protocol was approved by the Institutional Review Board (IRB) of EMPHNET on September 1, 2025 (Protocol No. 2025/9/E6), and all procedures were conducted in accordance with applicable ethical standards. The study involved minimal risk and focused on professional expertise rather than personal or sensitive data. Participation was voluntary, informed consent was implied through survey completion, and findings were reported in aggregate form only.

### Data analysis

Quantitative data from Delphi Round 2 were analyzed using descriptive statistics, including means, standard deviations, frequencies, and percentages. Qualitative data from Delphi Round 1 were summarized thematically and used exclusively to inform the development of Round 2 items. Data were managed and analyzed using standard spreadsheet and statistical software.

## Results

### Panel characteristics and response

A total of 30 experts participated in Delphi Round 2, with the majority also having participated in Round 1. Participants were based in 13 countries and represented a wide range of professional roles and disciplinary backgrounds. Most panelists were based in the Eastern Mediterranean Region, including Jordan (*n* = 5), Palestine (*n* = 3), Morocco (*n* = 2), and one each from Egypt, Iraq, Yemen, Iran, and Afghanistan; Pakistan (*n* = 6) contributed the largest single-country group. Additional participants were based in the United Kingdom (*n* = 6) and one each in the United States, Ghana, and Bangladesh, reflecting the involvement of international technical partners alongside regional experts. Half of respondents (50.0%) reported serving as FETP Directors, Resident Advisors, Coordinators, or Faculty. Panelists reported multidisciplinary expertise, most commonly in epidemiology (90.0%) and environmental health (70.0%), followed by field epidemiology (56.7%), climate change (53.3%), and public health policy and planning (53.3%). Additional areas of expertise included toxicology, International Health Regulations/Global Health Security, surveillance and laboratory systems, chemical surveillance and risk assessment, and public health emergency management. This breadth of expertise supported a comprehensive assessment of competencies spanning scientific, operational, and policy perspectives (Table 1).

**Table 1:** Characteristics and areas of expertise of experts participating in Delphi Round 2 (*n* = 30).

Area of expertise	*n*	%
Core public health expertise		
Epidemiology	27	90.0
Environmental health	21	70.0
Field epidemiology	17	56.7
Public health policy & planning	16	53.3
Climate change	16	53.3
Specialized and operational expertise		
International Health regulations / global health security (IHR/GHS)	7	23.3
Rapid response teams (RRT)	4	13.3
Surveillance and laboratory systems	4	13.3
Toxicology	4	13.3
Public Health Emergency Management (PHEM)	2	6.7
Chemical surveillance, risk assessment, and incident response	1	3.3

### Delphi round 2

Across all competency domains, expert ratings were consistently high and mostly ≥4.0 on a 5-point Likert scale, indicating strong consensus for inclusion of climate change and environmental epidemiology competencies within FETP training. Across the 38 rated competencies, mean importance ratings ranged from 3.77 to 4.83, and the great majority of items (32 of 38) had a mean ≥4.0. The proportion of respondents assigning a rating of ≥4 ranged from 56.7 to 100%, and the interquartile range was ≤1 point for most items, indicating limited dispersion around consistently high ratings. Item-level proportions of ratings ≥4 and IQRs are reported alongside means and standard deviations in Table 2. The highest levels of consensus were observed for competencies that extend core field epidemiology functions—particularly surveillance, outbreak investigation, data analysis, and multisectoral collaboration—into climate and environmental contexts. These highest-consensus competencies constitute a compact, high-priority subset recommended for immediate integration at the Frontline/Basic level, whereas the fuller competency set is best used as a tiered menu across the Basic, Intermediate, and Advanced levels. For immediate Basic/Frontline integration, the highest-priority subset includes basic environmental epidemiology concepts, identification of major environmental hazards and exposure pathways, climate-sensitive disease surveillance, incorporation of climate and environmental indicators into surveillance, outbreak investigation with environmental or climate drivers, basic exposure assessment, risk communication, and multisectoral/One Health coordination.

**Table 2:** Expert ratings and consensus on climate change and environmental epidemiology competencies for integration into field epidemiology training programs (*n* = 30)*.

Competency	Mean (SD)	Median (IQR)	% rated ≥4	Included in FETP, n (%)
Foundational knowledge				
Fundamentals of environmental epidemiology (concepts, study designs, exposure–outcome relationships).	4.83 (0.38)	5.0 (5.0–5.0)	100.0%	30 (100.0)
Environmental hazards, sources, and pathways to exposure	4.63 (0.62)	5.0 (4.0–5.0)	93.3%	30 (100.0)
Understanding the interlinkages between climate and environmental change, environmental hazards, and their impacts on population health.	4.53 (0.73)	5.0 (4.0–5.0)	86.7%	28 (93.3)
Key environmental health determinants (air, water, soil, ecosystem factors).	4.33 (0.80)	4.5 (4.0–5.0)	86.7%	28 (93.3)
Understanding climate science and environmental processes (e.g., greenhouse effect, climate systems).	4.23 (0.77)	4.0 (4.0–5.0)	80.0%	28 (93.3)
Environmental health determinants (socioeconomic, political, developmental, infrastructural).	4.23 (0.86)	4.0 (4.0–5.0)	80.0%	26 (86.7)
Basics of toxicology: types of substances, exposure pathways, absorption, metabolism, and dose–response.	3.93 (1.08)	4.0 (3.0–5.0)	66.7%	25 (83.3)
Environmental health exposures and risk assessment				
Identification of major environmental hazards (biological, chemical, physical, radiological).	4.70 (0.47)	5.0 (4.0–5.0)	100.0%	29 (96.7)
Approaches to exposure assessment for epidemiologic and population-based studies (measuring air, water, soil contaminants).	4.57 (0.68)	5.0 (4.0–5.0)	90.0%	29 (96.7)
Environmental and occupational health risk assessment (quantifying health risks from exposures).	4.27 (0.91)	4.5 (4.0–5.0)	83.3%	28 (93.3)
Health impact assessment of toxic exposures (acute and chronic).	4.03 (0.93)	4.0 (3.2–5.0)	73.3%	24 (80.0)
Application of toxicology principles to exposure pathways and health outcomes.	3.77 (1.01)	4.0 (3.0–5.0)	56.7%	23 (76.7)
Epidemiological and analytical skills				
Study design to examine the link between environmental hazard and health outcome.	4.83 (0.38)	5.0 (5.0–5.0)	100.0%	29 (96.7)
Data linkage and analysis to quantify the association between exposure and health outcome.	4.53 (0.68)	5.0 (4.0–5.0)	90.0%	28 (93.3)
Estimate the health impacts of climate change-related or non-related exposure.	4.43 (0.82)	5.0 (4.0–5.0)	86.7%	26 (86.7)
Applying GIS and Remote Sensing (satellite) data for environmental health monitoring.	4.03 (0.85)	4.0 (3.0–5.0)	66.7%	25 (83.3)
Surveillance and outbreak response				
Applying outbreak investigation methods to events with environmental/climate factors.	4.63 (0.72)	5.0 (4.2–5.0)	93.3%	29 (96.7)
Monitoring and responding to climate-sensitive conditions (vector-borne, water-borne, heat-related).	4.50 (0.78)	5.0 (4.0–5.0)	90.0%	29 (96.7)
Integrating climate/environmental indicators into disease surveillance systems.	4.43 (0.86)	5.0 (4.0–5.0)	90.0%	26 (86.7)
Coordinating outbreak investigations with environmental/climate components.	4.43 (0.90)	5.0 (4.0–5.0)	86.7%	29 (96.7)
Using early warning systems and climate forecasts to anticipate health threats.	4.20 (0.89)	4.0 (4.0–5.0)	83.3%	28 (93.3)
Vulnerability, adaptation and preparedness				
Understanding socioeconomic and societal determinants of environmental health.	4.20 (1.00)	4.5 (4.0–5.0)	76.7%	27 (90.0)
Conducting vulnerability and adaptation (V&A) assessments for environmental health risks.	4.00 (1.29)	4.0 (4.0–5.0)	76.7%	23 (76.7)
Preparedness and response to toxicological emergencies (industrial accidents, pollution events).	3.87 (1.17)	4.0 (3.0–5.0)	66.7%	24 (80.0)
Strengthening health system resilience to climate hazards.	3.87 (1.20)	4.0 (3.2–5.0)	73.3%	24 (80.0)
Designing and evaluating adaptation/resilience strategies (e.g., heat plans, flood mitigation).	3.83 (1.23)	4.0 (3.2–5.0)	73.3%	22 (73.3)
Policy, leadership and governance				
Incorporating climate/environmental considerations into public health planning and leadership.	4.33 (0.92)	5.0 (4.0–5.0)	83.3%	25 (83.3)
Promoting ethical and equity-oriented approaches to decision-making.	4.10 (0.96)	4.0 (3.2–5.0)	73.3%	23 (76.7)
Advocating for resilience, equity, and climate justice in health policy.	4.00 (1.02)	4.0 (3.0–5.0)	70.0%	21 (70.0)
Understanding global/national climate and health policies and frameworks (Paris Agreement, SDGs, national plans).	3.97 (1.13)	4.0 (3.0–5.0)	63.3%	24 (80.0)
Risk communication and community engagement				
Developing effective communication strategies for environmental and climate-related health risks.	4.40 (0.97)	5.0 (4.0–5.0)	83.3%	26 (86.7)
Community engagement in adaptation and prevention efforts.	4.27 (0.94)	4.5 (4.0–5.0)	83.3%	25 (83.3)
Risk communication to policymakers, stakeholders, and communities regarding toxic and environmental exposures.	4.27 (1.02)	5.0 (4.0–5.0)	83.3%	27 (90.0)
Participating in public education/awareness on environmental health issues.	4.23 (0.97)	4.0 (4.0–5.0)	86.7%	23 (76.7)
One Health and intersectoral collaboration				
Engaging in multisectoral planning/partnerships (water, agriculture, transport, construction, food systems, urban planning).	4.53 (0.97)	5.0 (4.2–5.0)	90.0%	27 (90.0)
Applying the One Health approach (human–animal–ecosystem).	4.50 (1.01)	5.0 (4.2–5.0)	86.7%	29 (96.7)
Collaborating with the veterinary, agricultural, and environmental sectors.	4.33 (0.96)	5.0 (4.0–5.0)	83.3%	25 (83.3)
Integrating animal/environmental surveillance into public health monitoring.	4.23 (1.10)	5.0 (4.0–5.0)	80.0%	26 (86.7)

*Ratings were made on a 5-point Likert importance scale (1 = lowest, 5 = highest). “% rated ≥4” is the proportion of respondents assigning an importance score of 4 or 5; “Median (IQR)” reports the median and interquartile range (25th–75th percentile). Consensus criteria were pre-specified.

### Foundational knowledge

Strong consensus was achieved for foundational competencies underpinning climate change and environmental epidemiology. The highest-rated competency was fundamentals of environmental epidemiology, including concepts, study designs, and exposure–outcome relationships (mea*n* = 4.83, SD = 0.38), with 100% of respondents endorsing inclusion in FETP curricula. High importance was also assigned to environmental hazards, sources, and exposure pathways (mea*n* = 4.63), and to understanding interlinkages between climate change, environmental hazards, and population health impacts (mea*n* = 4.53).

Competencies related to key environmental health determinants (air, water, soil, and ecosystem factors), climate science and environmental processes, and broader socioeconomic and infrastructural determinants of environmental health were also rated highly (means ranging from 4.23 to 4.33). Competencies related to basic toxicology received comparatively lower, though still substantial, ratings (mea*n* = 3.93), with more than 80% of respondents supporting inclusion. These foundational competencies underpin multiple subsequent domains, including surveillance, outbreak investigation, and analytical skills.

### Environmental health exposures and risk assessment

Experts reached a strong consensus on competencies related to hazard identification and exposure assessment. Identification of major environmental hazards (biological, chemical, physical, and radiological) received a mean rating of 4.70, while approaches to exposure assessment for epidemiologic and population-based studies scored 4.57. Environmental and occupational health risk assessment was also rated highly (mea*n* = 4.27).

Competencies related to health impact assessment of toxic exposures and application of toxicology principles received slightly lower mean ratings (means 3.77–4.03) and lower inclusion percentages compared with other domains, though the majority of respondents still supported their inclusion. Toxicology-related competencies were framed within the scope of applied field epidemiology, focusing on the interpretation of exposure pathways and health impacts rather than specialist laboratory analysis.

### Epidemiological and analytical skills

A very strong consensus emerged for epidemiological and analytical competencies. Study design to examine associations between environmental hazards and health outcomes was among the highest-rated items overall (mea*n* = 4.83). Data linkage and analytical skills to quantify exposure–outcome relationships also received high ratings (mea*n* = 4.53), as did competencies related to estimating health impacts of climate-related exposures (mea*n* = 4.43).

Competencies involving the application of geographic information systems (GIS) and remote sensing data for environmental health monitoring were rated slightly lower but remained strongly supported (mea*n* = 4.03), with more than 80% of respondents endorsing inclusion. These competencies operationalize foundational knowledge within routine FETP field investigations and analyses.

### Surveillance and outbreak response

Consensus was particularly strong for competencies directly aligned with core FETP practice. Applying outbreak investigation methods to events with environmental or climate drivers achieved a mean rating of 4.63, while monitoring and responding to climate-sensitive conditions, including vector-borne, water-borne, and heat-related illnesses, scored 4.50.

Integrating environmental and climate indicators into disease surveillance systems and coordinating outbreak investigations with environmental and climate components were also highly rated (mean 4.43). Use of early warning systems and climate forecasts to anticipate health threats received a mean rating of 4.20, reflecting strong support for predictive and preparedness-oriented competencies. Overall, these competencies received some of the highest levels of consensus across domains.

### Vulnerability, adaptation, and preparedness

Competencies related to vulnerability and adaptation demonstrated moderate-to-high consensus. Understanding socioeconomic and societal determinants of environmental health received a mean rating of 4.20. Conducting vulnerability and adaptation assessments, preparedness for chemical emergencies, strengthening health system resilience to climate hazards, and designing adaptation strategies received mean ratings ranging from 3.83 to 4.00, with some variability across respondents. Respondents in Delphi round 1 generally viewed these competencies as more appropriate for Intermediate and Advanced FETP levels, given their resource requirements and systems-level orientation.

### Policy, leadership, and governance

Respondents rated competencies related to policy and leadership as highly important. Incorporating climate and environmental considerations into public health planning and leadership achieved a mean rating of 4.33. Ethical and equity-oriented decision-making, advocacy for resilience and climate justice, and understanding global and national climate–health frameworks all received mean ratings around 4.0, with inclusion percentages ranging from 70.0 to 83.3%.

### Risk communication and community engagement

High consensus was observed for risk communication and community engagement competencies. Developing effective communication strategies for environmental and climate-related health risks achieved a mean rating of 4.40. Risk communication to policymakers, stakeholders, and communities regarding toxic and environmental exposures scored 4.27, while community engagement in adaptation and prevention efforts and public education activities were also strongly supported.

### One Health and Intersectoral collaboration

Competencies related to One Health and multisectoral collaboration received some of the highest overall ratings. Engaging in multisectoral planning and partnerships across sectors such as water, agriculture, transport, construction, food systems, and urban planning achieved a mean rating of 4.53. Applying the One Health approach scored 4.50, with nearly all respondents supporting inclusion. Integration of animal and environmental surveillance into public health monitoring and collaboration with veterinary and environmental sectors were also rated highly. These competencies were consistently framed in terms of coordination and collaboration for surveillance and outbreak response, rather than sector-specific technical roles.

### Validation and refinement of competencies through the round 3 workshop

Workshop participants unanimously endorsed the core competency domains identified in Delphi Round 2. Discussions focused on clarifying terminology, reducing overlap between domains, and ensuring alignment with applied field epidemiology practice. No competency domains were removed; however, minor refinements were made to improve clarity and operational relevance, particularly for surveillance integration, exposure assessment, and multisectoral coordination.

Participants emphasized that competencies should be implemented in a tiered manner across FETP levels. Foundational competencies related to surveillance, outbreak investigation, and basic exposure assessment were considered most appropriate for Basic FETP, while advanced analytical, policy, and adaptation competencies were more suitable for Intermediate and Advanced training levels.

### Existing FETP competencies adaptable to climate and environmental health

Rather than introducing entirely new curricular domains, respondents in Delphi round 1 emphasized adaptation of existing FETP competencies. Surveillance (89.7%), outbreak investigation (82.8%), and data analysis and interpretation (79.3%) were most frequently identified as adaptable areas. Respondents highlighted the integration of environmental indicators, meteorological data, toxicological monitoring, and climate-sensitive disease trends into these established competencies. Risk communication, policy and advocacy, research methods, and leadership and multisectoral collaboration were also commonly identified as adaptable domains.

### Field work activities for FETP training

Respondents in Round 1 proposed a range of field-based activities to strengthen practical skills in environmental epidemiology, toxicology, and climate-related health (Table 3). The most frequently recommended areas were climate-sensitive disease surveillance (86.2%), exposure and risk assessment (79.3%), chemical and radiological incident response (72.4%), outbreak investigation with environmental drivers (69.0%), and risk communication and community engagement (69.0%).

**Table 3:** Recommended field work activities for FETP trainees in environmental epidemiology, toxicology, and climate-related health (*n* = 29).

Technical area	Specific topics mentioned	*n* (%)
Climate-sensitive disease surveillance	Surveillance of vector-, water-, food-borne, zoonotic, and heat-related illnesses; syndromic and sentinel surveillance; integration of climate and environmental indicators (temperature, rainfall, air quality, pollution).	25 (86.2%)
Exposure and risk assessment	Environmental measurement and monitoring (air, water, soil, food contamination); exposure pathways and dose–response relationships; vulnerability and adaptation assessment; quantitative and qualitative risk analysis; development of mitigation strategies.	23 (79.3%)
Chemical and radiological incident response	Investigation of toxic substance exposures (chemical spills, pesticide poisoning, industrial pollution); principles of environmental toxicology; radiological, occupational, and hazardous waste exposures; preparedness and response to toxicological emergencies.	21 (72.4%)
Outbreak investigation with environmental drivers	Field investigations of outbreaks or events with environmental drivers, including floods, droughts, contamination events, and vector surges; disaster epidemiology; post-disaster health impact and needs assessments.	20 (69.0%)
Risk communication and community engagement	Risk communication for environmental and toxicological threats (chemical spills, heatwaves, floods, vector control); community engagement strategies; multisectoral messaging in emergencies; addressing misinformation.	20 (69.0%)
GIS and spatial epidemiology	Mapping exposures and disease hotspots; integrating meteorological, environmental, and toxicological data; applying remote sensing and spatial modeling for vulnerability and risk assessment.	18 (62.1%)
Disaster risk management and emergency response	Preparedness and response to floods, droughts, heatwaves, wildfires, and industrial accidents; strengthening health system resilience; early warning systems; mental health and psychosocial support in disasters.	17 (58.6%)
One Health and multisectoral collaboration	Applying One Health for human–animal–environment interactions; cross-sector coordination with agriculture, environment, water, and disaster management; integrating ecosystem and environmental health into surveillance and preparedness.	15 (51.7%)
Policy, governance and health systems	Policy advocacy for environmental health and climate resilience; adaptation frameworks (NAPs, SDGs, national climate strategies); governance for toxicological risk management; sustainable and climate-resilient health facilities.	13 (44.8%)
Regional priorities	WASH and food hygiene; water and wastewater management and surveillance; industrial and urban air pollution and respiratory health; nutrition and food security under climate stress; displacement and health in fragile contexts.	12 (41.4%)

These activities were intended to illustrate applied learning approaches rather than prescribe a uniform set of field exercises.

A key outcome of the Round 3 workshop was the translation of validated competencies into applied learning modalities. Participants proposed a set of regionally relevant teaching case studies reflecting real public health events, including chemical explosions and spills, floods, heatwaves, industrial pollution, air quality crises, and climate-sensitive disease outbreaks. These case studies were designed to integrate multiple competency domains, such as surveillance, outbreak investigation, exposure assessment, risk communication, and governance.

In addition, participants proposed structured fieldwork activities aligned with routine FETP placements. Suggested activities included integrated surveillance system assessments, environmental and climate risk assessments, industrial site hazard visits, ecological analyses linking climate variables to health outcomes, and development of risk communication products. These activities were intended to produce tangible public health outputs while reinforcing competency development through supervised practice.

Table 4 shows a matrix linking representative competencies to FETP level, learning activity, and expected field output. “EMR priority” indicates competencies especially relevant to Eastern Mediterranean Region hazards (extreme heat, water insecurity, air pollution, WASH disruption, chemical incidents, conflict-related contamination, and climate-sensitive vector- and water-borne diseases). The list is illustrative rather than exhaustive; “Basic” competencies are recommended for immediate integration, with Intermediate and Advanced competencies phased in as capacity allows.

**Table 4:** Illustrative matrix linking representative competencies to FETP level, learning activity, and expected field output.

Representative competency	FETP level	Illustrative learning activity	Expected field output	EMR priority
Climate-sensitive disease surveillance (vector-, water-, heat-related)	Basic	Add temperature, rainfall, and air-quality indicators to an existing surveillance dataset and flag anomalies	Short surveillance brief linking a climate signal to a notifiable condition	Yes
Applying outbreak investigation methods to environmental/climate events	Basic	Extend a routine outbreak investigation to include an environmental exposure hypothesis	Investigation report with an environmental exposure assessment	Yes
Environmental sampling and exposure assessment	Intermediate	Field exercise collecting and interpreting water or air samples during a simulated incident	Exposure assessment memo with recommended control measures	Yes
Vulnerability and adaptation (V&A) assessment	Intermediate	Conduct a district-level heat or WASH vulnerability assessment	V&A report identifying priority populations and adaptation actions	Yes
Toxicological emergency and chemical-incident response	Intermediate	Tabletop exercise on an industrial or conflict-related chemical release	Rapid risk assessment and coordinated response plan	Yes
Climate- and environment-informed policy and governance	Advanced	Draft a policy brief integrating climate-health evidence into a national adaptation plan	Policy brief for ministry of health/environment stakeholders	Yes
One Health and multisectoral coordination	Advanced	Design a cross-sector surveillance protocol with environmental and veterinary partners	Multisectoral protocol and data-sharing agreement	Yes
Risk communication on environmental and chemical hazards	Advanced	Develop and pilot risk-communication materials for an at-risk community	Tested risk-communication package and evaluation notes	Yes

### Barriers to institutionalizing a climate and environmental health module

All 29 respondents in Delphi round 1 identified barriers to institutionalizing a climate and environmental health module within FETPs. Barriers were grouped into institutional and governance-related, resource-related, technical and operational, and human resource constraints.

Institutional and governance-related barriers were most frequently reported (86.2%), including lower prioritization of climate and environmental health relative to infectious diseases or noncommunicable diseases and weak intersectoral coordination among health, environment, and meteorology sectors. Resource-related barriers were reported by 82.8% of respondents and included a lack of dedicated funding, dependence on external donors, and high costs of equipment and field-based training.

Technical and operational barriers were identified by 22 respondents (75.9%), particularly limited access to reliable environmental and climate data, insufficient laboratory capacity for toxicological investigations, and the absence of standardized curricula and tools. Human resource barriers were mentioned by 21 respondents (72.4%), including shortages of trainers with combined epidemiology and climate expertise, competing trainee workloads, limited mentorship capacity, and concerns about staff turnover or “brain drain.” A smaller number of respondents also cited regional challenges such as political instability, sanctions, and conflict.

### Enablers and opportunities for adoption

All 29 respondents in round 1 identified enablers to support institutionalization. Policy and institutional momentum (86.2%) and regional or global collaboration platforms (82.8%) were most frequently cited. Respondents highlighted global policy commitments, national adaptation plans, and existing regional FETP infrastructure—supported by organizations such as EMPHNET and WHO—as key entry points for adoption. Financial opportunities were noted by 22 respondents (75.9%), including blended funding and alignment with preparedness and resilience priorities. Technical enablers were reported by 20 respondents (69.0%), reflecting increased availability of open-access datasets, digital tools, and established methodological frameworks. Human resource and mentorship opportunities were identified by 19 respondents (65.5%), emphasizing regional expertise, alumni networks, and train-the-trainer approaches. Contextual urgency driven by the burden of climate-sensitive health problems was noted by 15 respondents (51.7%).

### Key stakeholders for module development and implementation

Respondents in Round 1 identified a broad range of stakeholders necessary for effective development and institutionalization. National stakeholders were most frequently cited (27 of 29; 93.1%), with Ministries of Health identified as primary leaders for policy alignment and curriculum integration. Other national actors included Ministries of Environment, Meteorology, Agriculture, and Water, as well as universities, research centers, and national public health institutes.

Regional stakeholders were identified by 24 respondents (82.8%), particularly regional FETP networks and WHO Regional Offices. Global stakeholders were cited by 22 respondents (75.9%) and included WHO, US CDC, UKHSA, UN agencies, the World Bank, and donor organizations. Civil society, NGOs, and private sector actors were noted by 9 respondents (31.0%).

## Discussion

This Delphi study provides a consensus-based framework of core competencies in climate change and environmental epidemiology for integration into FETPs. Through a structured, three-round process, experts from diverse geographic and professional backgrounds converged on a set of competencies that reflect both the expanding scope of environmental and climate-related health risks and the operational realities of applied field epidemiology. The consistently high ratings across domains—particularly for surveillance, outbreak investigation, data analysis, and multisectoral collaboration—underscore broad agreement that climate change and environmental hazards are not peripheral concerns, but integral to routine epidemiologic practice.

Returning to the research questions posed at the outset, this study identifies a comprehensive set of core competencies in climate change and environmental epidemiology (question 1), maps them across the Basic, Intermediate, and Advanced FETP levels (question 2), and—through the Round 3 workshop—translates them into teaching case studies and structured fieldwork suitable for existing FETP curricula (question 3). In relation to existing frameworks, the competencies identified here both align with and extend established resources. They map closely onto the domains of the WHO and US CDC FETP core competencies—particularly surveillance, outbreak investigation, and data analysis—and onto broader climate–health competency sets such as the Global Consortium on Climate and Health Education (GCCHE) core competencies and WHO guidance on climate-resilient health systems. Whereas these existing frameworks are generally oriented toward academic or clinical training, our framework is distinctive in embedding climate and environmental content within the applied, service-oriented FETP model and in explicitly foregrounding chemical hazards, conflict-related environmental exposures, and multisectoral response priorities that are especially salient in the EMR. In particular, the competencies most distinctly EMR-informed are those addressing extreme heat exposure and heat–health early-warning systems; water insecurity and WASH disruption; ambient and household air pollution; surveillance of climate-sensitive vector-borne and water-borne diseases (e.g., dengue, leishmaniasis, cholera); chemical incidents and toxicological emergencies; and conflict- and displacement-related environmental contamination. Foregrounding these priorities is what differentiates the framework from more globally oriented climate–health competency sets and anchors it in the specific hazard profile of the region.

The competencies identified align closely with global guidance on climate-resilient health systems and public health workforce development. International frameworks emphasize the need to move beyond awareness of climate risks toward operational capacity for climate-informed surveillance, early warning, preparedness, and response (12). The strong consensus observed for competencies related to exposure assessment, climate-sensitive surveillance, early warning systems, and risk communication mirrors priorities highlighted in the *Lancet Countdown on Health and Climate Change*, which documents persistent gaps between escalating climate-related health risks and public health preparedness (13).

Importantly, the highest-rated competencies were those most closely aligned with established FETP functions—surveillance, outbreak investigation, and applied data analysis—suggesting that experts view climate change and environmental hazards as extensions of core epidemiologic practice rather than as distinct or specialized domains. This finding is consistent with calls in the epidemiologic literature for integrated, systems-oriented approaches to complex and interacting health risks (9).

The relevance of these findings is particularly pronounced for the EMR, where climate change, environmental degradation, chemical hazards, conflict, and displacement converge to produce complex and compounding health risks. Regional assessments highlight air pollution, unsafe water, chemical exposures, extreme heat, and climate-sensitive infectious diseases as major contributors to preventable morbidity and mortality (14). The strong emphasis placed by respondents on One Health and multisectoral collaboration reflects the operational realities faced by epidemiologists in the EMR, where effective responses often require coordination across health, environment, agriculture, water, meteorology, and disaster management sectors.

The identified barriers—particularly institutional prioritization, financing constraints, limited technical capacity, and shortages of trained mentors—are consistent with broader analyses of health system capacity for climate adaptation in low- and middle-income and conflict-affected settings (8). At the same time, respondents highlighted substantial enablers, including policy momentum, existing regional FETP infrastructure, growing availability of open data and digital tools, and strong regional and global partnerships. Together, these findings suggest that while challenges to institutionalization are significant, conditions for adoption are increasingly favorable.

Across both Delphi rounds, experts consistently emphasized integration rather than expansion of curricula. Rather than creating stand-alone climate or environmental health modules that compete with existing priorities, respondents favored embedding climate- and environment-related content within existing FETP competencies, including surveillance, outbreak investigation, data analysis, and risk communication. This approach aligns with broader calls for integrated curriculum design to address training overload while maintaining relevance to evolving public health threats (15). Embedding climate-related scenarios into routine field projects and case studies may also facilitate institutional ownership and sustainability, particularly in resource-constrained settings.

The consensus-based competency framework generated by this study provides a practical foundation for aligning FETP curricula with national climate–health strategies, National Adaptation Plans, and global commitments under the Paris Agreement and the Sustainable Development Goals. For ministries of health and national public health institutes, adoption of these competencies can strengthen integration of climate and environmental considerations into routine surveillance, outbreak response, and preparedness planning. More broadly, equipping the field epidemiology workforce with these competencies contributes to strengthening environmental public health (EPH) services and practice, complementing wider workforce development and capacity-building efforts across the EPH system and reinforcing the competencies that such services depend upon (16).

Evidence from emergency health-system reorganization and healthcare cleaning and disinfection practices highlights the importance of resilience, prevention, and environmental safety during public health emergencies (17, 18). These experiences support strengthening field epidemiology competencies in preparedness, hazard identification, risk assessment, and intersectoral coordination.

At the programmatic level, FETP implementers can use the framework to guide curriculum revision, prioritize faculty development, and design field-based learning activities that reflect real-world climate and environmental challenges. Regional FETP networks and partners are well-positioned to support harmonization, peer learning, and resource mobilization across countries.

The inclusion of a validation and operationalization workshop strengthens this study by addressing a key limitation of Delphi-based competency development—namely, the gap between consensus-based frameworks and practical implementation. While Delphi methods are effective for prioritizing expert opinion, they may not fully capture feasibility within real training and public health system constraints. The Round 3 workshop enabled experts to contextualize competencies within diverse FETP settings, validate their applicability across countries, and translate them into concrete learning approaches, including case-based learning and supervised fieldwork.

Although the Round 3 workshop enhanced contextual validity and implementation relevance, it relied on qualitative consensus rather than quantitative re-scoring of competencies. This approach may limit formal measurement of agreement; however, it was appropriate given the workshop’s primary objective of validation, refinement, and operational translation rather than reprioritization. Moreover, the workshop generated a rich and diverse discussion of country- and community-specific needs and of the development, implementation, and sustainability of such training, strengthening the contextual grounding of the framework and supporting its transferability and adaptation across different settings. Future studies may consider mixed quantitative-qualitative approaches to further assess implementation outcomes. Although the panel included diverse public health, academic, FETP, and policy expertise, some non-health sectors—particularly meteorology, environmental regulation, agriculture, water, civil society, and community implementation partners—were less represented in the survey rounds. Their perspectives were partly incorporated during the validation workshop and should be expanded in future implementation work.

In conclusion, this study provides one of the first Delphi-based, consensus-driven frameworks for integrating climate change and environmental epidemiology competencies into FETP curricula. The findings demonstrate strong agreement among experts that these competencies are central to applied epidemiology practice, particularly in regions facing intersecting climate, environmental, and chemical hazards. By emphasizing integration into existing FETP structures, tiered competency development, and applied, context-specific learning, the framework offers a feasible and scalable pathway for strengthening climate-resilient epidemiologic capacity. The barriers identified by respondents—limited prioritization and funding, shortages of trainers with combined climate and epidemiology expertise, weak intersectoral coordination, and constrained access to environmental data—point to specific, feasible implementation strategies. Rather than creating a stand-alone course, competencies can be embedded incrementally into existing FETP activities, for example by adding climate and environmental dimensions to routine outbreak-investigation and surveillance-evaluation exercises. Trainer shortages can be mitigated through train-the-trainer models, regionally shared and modular online curricula, and alumni and mentorship networks that pool scarce expertise across countries. Weak intersectoral coordination and data gaps can be addressed through formal partnerships with meteorological and environmental agencies to enable data sharing and joint exercises. Finally, adoption can be phased according to program capacity—beginning with the high-priority Frontline/Basic competencies and lightweight activities feasible in low-resource and conflict-affected settings, and expanding to Intermediate and Advanced competencies as resources allow. Institutionalizing these competencies within FETPs can contribute to more responsive surveillance systems, improved preparedness, and more effective public health action in the face of increasingly complex and interconnected health threats.

## Data Availability

The raw data supporting the conclusions of this article will be made available by the authors, without undue reservation.

## References

[1] Prüss-Üstün A Wolf J Corvalán CF Bos R Neira MP. Preventing Disease through Healthy Environments: a Global Assessment of the Burden of Disease from Environmental Risks. Geneva: World Health Organization (2016).

[2] Health Effects Institute, Institute for Health Metrics and Evaluation. State of Global Air 2025: A Report on Air Pollution and its Role in the World's Leading Causes of Death. Boston, MA: Health Effects Institute (2025).

[3] Alzayadneh EM Alhadidi KA Alasasfeh I Battah A Khasawneh SM Faouri MN . General pattern of paediatric poisoning in Jordan during 2018–2019. Toxicol Rep. (2024) 12:369–74. doi: 10.1016/j.toxrep.2024.03.008, 38572466 PMC10987798

[4] Mostafalou S Abdollahi M. Pesticides: an update of human exposure and toxicity. Arch Toxicol. (2017) 91:549–99. doi: 10.1007/s00204-016-1849-x, 27722929

[5] World Health Organization. Chemical Incidents. Geneva: World Health Organization.

[6] Intergovernmental Panel on Climate Change. Climate change 2022: Impacts, Adaptation and Vulnerability. Contribution of Working Group II to the Sixth Assessment Report of the IPCC. Cambridge: Cambridge University Press (2022).

[7] Romanello M Napoli CD Green C Kennard H Lampard P Scamman D . The 2023 report of the lancet countdown on health and climate change: the imperative for a health-centred response in a world facing irreversible harms. Lancet. (2023) 402:2346–94. doi: 10.1016/S0140-6736(23)01859-7, 37977174 PMC7616810

[8] Ebi KL Semenza JC Rocklöv J. Current medical research funding and frameworks are insufficient to address the health risks of global environmental change. Environ Health. (2016) 15:108. doi: 10.1186/s12940-016-0183-3, 27835959 PMC5106817

[9] Semenza JC Rocklöv J Ebi KL. Climate change and cascading risks from infectious disease. Infect Dis Ther. (2022) 11:1371–90. doi: 10.1007/s40121-022-00647-3, 35585385 PMC9334478

[10] Jones DS Dicker RC Fontaine RE Boore AL Omolo JO Ashgar RJ . Building global epidemiology and response capacity with field epidemiology training programs. Emerg Infect Dis. (2017) 23:S158–65. doi: 10.3201/eid2313.170509, 29155658 PMC5711325

[11] Polonsky JA Baidjoe A Kamvar ZN Cori A Durski K Edmunds WJ . Outbreak analytics: a developing data science for informing the response to emerging pathogens. Philos Trans R Soc Lond Ser B Biol Sci. (2019) 374:20180276. doi: 10.1098/rstb.2018.0276, 31104603 PMC6558557

[12] Intergovernmental Panel on Climate Change. Climate change 2023: Synthesis Report—Summary for Policymakers. Geneva: IPCC (2023).

[13] Romanello M Walawender M Hsu SC Moskeland A Palmeiro-Silva Y Scamman D . The 2025 report of the lancet countdown on health and climate change: climate change action offers a lifeline. Lancet. (2025) 406:2804–57. doi: 10.1016/S0140-6736(25)01919-1, 41175887

[14] World Health Organization Regional Office for the Eastern Mediterranean. Progress on the Health-Related Sustainable Development Goals and Targets in the Eastern Mediterranean Region. Cairo: WHO Regional Office for the Eastern Mediterranean (2023).

[15] Arora M Comrie AC Ernst KE. Assessing climate and health curriculum in graduate public health education in the United States. Front Public Health. (2023) 11:1124379. doi: 10.3389/fpubh.2023.1124379, 37139373 PMC10149657

[16] Leonardi GS Zeka A Ashworth M Bouland C Crabbe H Duarte-Davidson R . A new environmental public health practice to manage current and future global health challenges through education, training, and capacity building. Front Public Health. (2024) 12:1373490. doi: 10.3389/fpubh.2024.1373490, 39655257 PMC11627177

[17] Bianchi FP Cuscianna E Di Lorenzo A Daleno A Mele F Marra M . A new paradigm of hospital care for SARS-CoV-2 patients in the post-emergency phase in Italy. Ann Ig. (2023) 35:250–3. doi: 10.7416/ai.2022.2546 36222605

[18] Lattanzio S Cuscianna E Riformato G Noviello C Tafuri S Bianchi FP. Human and environmental impact of chemical agents used for cleaning, antisepsis, disinfection and sterilization in healthcare facilities. A semi-qualitative study among medical institutions of Puglia and Basilicata, southern Italy. Acta Biomed. (2025) 96:16146. doi: 10.23750/abm.v96i6.16146

